# From Waste to Styrene–Butadiene (SBR) Reuse: Developing PP/SBR/SEP Mixtures with Carbon Nanotubes for Antistatic Application

**DOI:** 10.3390/polym16172542

**Published:** 2024-09-08

**Authors:** Edson Duarte de Melo Sobrinho, Eduardo da Silva Barbosa Ferreira, Flávio Urbano da Silva, Elieber Barros Bezerra, Renate Maria Ramos Wellen, Edcleide Maria Araújo, Carlos Bruno Barreto Luna

**Affiliations:** 1 Academic Unit of Mechanical Engineering, Federal University of Campina Grande, Av. Aprígio Veloso, 882–Bodocongó, Campina Grande 58429-900, Paraíba, Brazil; 2 Academic Unit of Materials Engineering, Federal University of Campina Grande, Av. Aprígio Veloso, 882–Bodocongó, Campina Grande 58429-900, Paraíba, Brazilflavio.urbano@ifrn.edu.br (F.U.d.S.);; 3 Federal Institute of Education, Ciência e Tecnologia do Rio Grande do Norte, Natal 59015-000, Rio Grande do Norte, Brazil; 4 Department of Materials Engineering, Federal University of Paraíba, Cidade Universitária, João Pessoa 58051-900, Paraíba, Brazil

**Keywords:** styrene–butadiene, waste, reuse, carbon nanotubes, antistatic

## Abstract

Styrene–butadiene rubber (SBR) waste from the shoe industry was repurposed to produce polypropylene (PP)-based compounds, with the aim of evaluating their antistatic potential. Styrene–ethylene–propylene (SEP) was added as a compatibilizing agent, while carbon nanotubes (MWCNT) were incorporated as a conductive nanofiller. The polymer compounds were processed in an internal mixer, and injection molded. The properties evaluated included torque rheometry, melt flow index (MFI), impact strength, tensile strength, Shore D hardness, electrical conductivity, heat deflection temperature (HDT), and differential scanning calorimetry (DSC), along with scanning electron microscopy (SEM) for morphology analysis. The production of the PP/SBR/SEP (60/30/10 wt%) compound resulted in a ductile material, enhancing impact strength and elongation at break to 161.2% and 165.2%, respectively, compared to pure PP. The addition of SEP improved the compatibility of the PP/SBR system, leading to an increase in the torque curve and a reduction in the MFI. Furthermore, the SBR/SEP combination in PP accelerated the crystallization process and increased the degree of crystallinity, suggesting a nucleating effect. Carbon nanotubes, in concentrations ranging from 0.5 to 2 phr (parts per hundred resin), were added to the PP/SBR/SEP system. Only the PP/SBR/SEP/MWCNT compound with 2 phr of MWCNT was suitable for antistatic applications, exhibiting an electrical conductivity of 4.52 × 10^−07^ S/cm. This was due to the greater distribution of MWCNT in the PP matrix, as demonstrated by SEM. In addition, remains tough at room temperature, with a 166% increase in impact strength compared to PP. However, there was a reduction in elastic modulus, tensile strength, Shore D hardness, and HDT due to increased flexibility. SBR waste can be reintegrated into the production chain to produce antistatic polymeric compounds, obtaining a tough material at room temperature.

## 1. Introduction

The global rise in industrial product consumption has led to a significant increase in the generation of solid waste, which has, in turn, caused various environmental impacts [1,2]. This situation represents a growing challenge for waste management in large metropolises [3]. In this context, vulcanized synthetic rubbers require special attention due to increased post-consumer and post-industrial waste in natural ecosystems [4,5]. Generally, rubbers used in manufacturing undergo a vulcanization process, generating cross-links in the molecular structure [6]. As a result, vulcanized rubbers are highly durable, making their biological degradation difficult [7]. Rubbers are extensively used in the automotive and shoe industry, resulting in significant quantities of vulcanized elastomeric waste. This waste is a prominent environmental challenge [8,9]. The improper disposal of vulcanized elastomeric waste has garnered attention from society and the scientific community, who actively seek solutions for its effective treatment and disposal [10]. Therefore, recycling and reusing rubber are essential for environmental protection, conserving natural resources, and advancing the circular economy [11,12]. By implementing effective recycling practices, we can contribute to a more sustainable future and mitigate the environmental impact of rubber waste [13].

In 2020, global production of synthetic rubber was estimated at approximately 14.43 million tons, with styrene–butadiene rubber (SBR) being particularly prominent due to its excellent mechanical, elastic, and thermal properties, as well as its durability [14,15]. SBR is extensively used in tire and shoe manufacturing [16]. Substantial amounts of waste are generated both during production and in post-consumer disposal [17]. Reusing vulcanized SBR waste is extremely important, given that it is a raw material rich in additives to increase technical performance and useful life [18]. A viable method for recycling SBR is to use it as an impact modifier in polymers with low impact strength, such as polystyrene (PS), polyethylene (PE), and homopolymer polypropylene (PP) [19,20,21]. Incorporating SBR waste into the formulation of new products is economically advantageous and promotes the reuse of discarded materials, thereby adding value and benefiting the environment.

Polypropylene (PP) is a thermoplastic used in numerous industrial applications. This is due to its good mechanical properties, low density, chemical resistance, low water absorption, recyclability, excellent processability, and excellent cost-benefit [22,23]. Consequently, the literature [21,24] demonstrates that PP has been employed to develop polymer blends incorporating rubber waste. Ciro et al. [25] investigated polypropylene blends containing up to 55% by weight of waste rubber from the tire industry (WB). They observed that the torque curves for the PP/WB blends increased, particularly at higher rubber concentrations, indicating a rise in viscosity. Meanwhile, tensile strength and elastic modulus decreased with higher RBP content, while the elongation at break increased. Ong et al. [26] investigated the mechanical behavior of polypropylene (PP) blends with tire rubber waste (TRW), using up to 50 wt% TRW and various particle size ranges. They found that both the flexural elastic modulus and flexural strength decreased steadily with increasing TRW content, suggesting enhanced flexibility. Conversely, elongation at break and impact strength increased with the addition of TRW, with the most significant improvements observed at 40 wt% TRW. This was a critical concentration, since above 40% TRW, the elongation at break and impact strength declined. Overall, the mechanical properties were better when the TRW had the smallest particle size, specifically in the 250–500 µm range. Luna et al. [27] developed PP/shoe rubber waste (SRW) mixtures using styrene–(ethylene–butylene)–styrene (SEBS) as a compatibilizing agent, with concentrations of 20% and 30% styrene (St.). The addition of 30% SRW did not significantly affect the processability of PP. Incorporating 10% SEBS (with 20% St.) into the PP/SRW system resulted in a 316% increase in impact strength compared to pure PP. The elastic modulus, tensile strength, elongation at break, and Shore D hardness indicated greater flexibility in the PP/SRW/SEBS (20% St.) mixtures. Additionally, SEM analysis demonstrated SEBS effectiveness in compatibilizing the PP/SRW mixture, which is reflected in the improved mechanical properties.

Considering the specific review, rubber waste from the tire or shoe industry holds significant technological potential to enhance mechanical properties, thereby enabling the development of flexible materials tailor-made for particular applications. Improper disposal of rubber waste from the shoe industry results in significant environmental and social impacts [28]. Additionally, it is essential to reuse this rubber waste to avoid a loss of valuable raw material rich in additives and possessing desirable properties, representing an opportunity for its reintegration into the production chain. Therefore, environmental pollution and social aspects justify research seeking alternatives to promote the reuse of rubber waste, generating new materials with properties suitable for practical applications. This will contribute to sustainability and efficiency in the use of resources. Given the above, the specialized polymer literature did not mention producing polymer blends based on polypropylene with rubber waste from the shoe industry for antistatic materials, which is a novelty in the literature. Considering this, for the first time, styrene–butadiene (SBR) waste will be reused to manufacture flexible materials, using PP as the polymer matrix and aiming at the potential for antistatic application. To improve electrical conductivity, tailor-made materials will be developed using small amounts of carbon nanotubes (MWCNT). Antistatic polymeric materials are essential in various industries, as they protect sensitive electronic equipment, preventing fire and explosion risks [29,30]. Products with antistatic properties are designed to prevent the buildup of electrostatic charges. Examples include flexible covers, tweezers for handling electronic components, antistatic filters for dust removal in vacuum cleaners, supports for storing electronic products, panels for controlling static electricity, coatings for antistatic bags and transport cases, and coating films.

This research focused on producing polypropylene (PP) mixtures with shoe rubber waste (SBR), using styrene–ethylene–propylene (SEP) as a compatibilizer and carbon nanotubes (MWCNT) as conductive fillers. The potential of these compounds for use in antistatic packaging will be evaluated.

## 2. Materials and Methods

### 2.1. Materials

Polypropylene (PP), designated by the code H503, was used as the polymer matrix (Braskem, São Paulo, Brazil). This material was supplied by Braskem in the form of granules, with a density of 0.905 g/cm^3^ and a melt flow index of 3.5 g/10 min (ASTM D1238–230 °C/2.16 kg). Styrene–ethylene-propylene (SEP) copolymer, used as a compatibilizing agent, was supplied by Kraton under the commercial code G1701 (Kraton, Houston, TX, USA). This material, provided in powder form, contains 37% styrene (St.) and has a melt flow index (MFI) of less than 1 g/10 min (ASTM D1238–230 °C/5 kg). Vulcanized styrene–butadiene waste (SBR) from the shoe industry of São Paulo Alpargatas (Alpargatas S.A, Campina Grande, Brazil) was used as an impact modifier. This waste was supplied in powder form, with a black color. The SBR powder used was passed through a 60-mesh sieve (<250 µm). Multi-walled carbon nanotubes (MWCNTs) with 95% purity were used as conductive nanofillers (Advanced 2D, Beijing, China). Supplied by Advanced 2D Materials and produced via chemical vapor deposition (CVD), these MWCNTs have the following characteristics: lengths ranging from 3 to 12 µm, inner diameters of 3 to 5 nm, outer diameters of 8 to 15 nm, a specific surface area greater than 233 m^2^/g, and an electrical conductivity of 100 S/cm.

### 2.2. Processing and Molding

All pure materials were dried before processing to remove moisture, using a vacuum oven set at 60 °C for 24 h. Pure PP and PP/SBR/SEP/MWCNT mixtures were processed using a laboratory torque rheometer (Thermo Scientific Polylab QC, Waltham, MA, USA) with a capacity of 310 cm^3^ and roller rotors. Processing was performed under the following conditions: temperature of 190 °C, rotor speed of 60 rpm, and a residence time of 10 min. The operational parameters for processing were established based on the literature [31]. After processing, the material was ground in a knife mill to produce flakes suitable for injection molding. The detailed compositions are provided in Table 1, with proportions in weight percentages (%). The MWCNT nanofiller, used as a conductive additive, is expressed in parts per hundred of resin (phr).

The pure PP and the PP/SBR/SEP/MWCNT compounds were injection molded in an Arbug (Arburg Inc., Loßburg, Germany) injection molding machine (Allrounder 207C Golden Edition) to obtain impact, tensile, Shore D hardness, and HDT test specimens, according to the ASTM D256, ASTM D638, ASTM D2240, and ASTM D648 standards, respectively. The molding conditions of the test specimens were as follows:

- injection pressure: 1200 bar;
- temperature profile: 170 °C, 180 °C, 180 °C, 190 °C e 200 °C;
- mold temperature: 20 °C;
- mold cooling time: 25 s;
- holding pressure: 1000 bar.

The material processed in the internal mixer was molded into films with a thickness of 0.5 mm for electrical conductivity testing. A hydraulic press was employed at 190 °C for 4 min under a load of 5 tons. The films were then cooled to room temperature for 10 min while maintaining a load of 50 N. 

### 2.3. Characterization of SBR

The particle size distribution of SBR powder was determined according to the ASTM D5644 standard. For this purpose, a sieve shaker equipped with a set of overlapping sieves was used under vibration for 30 min.

Fourier-transform infrared spectroscopy (FTIR) was performed using a Bruker Alpha II spectrometer with the Attenuated Total Reflectance (ATR) method (Bruker Corporation, Billerica, EUA). The analysis was conducted on SBR waste in powder form, scanning in the range of 4000 to 400 cm^−1^ with a resolution of 4 cm^−1^ and 32 scans.

Energy-dispersive X-ray spectroscopy (EDX) was used to identify and quantify the chemical composition of the SBR powder. The analysis was performed using a Shimadzu EDX-720 system (Shimadzu Corporation, Kyoto, Japan).

Thermogravimetric analysis (TGA) was obtained from SBR powder in a Mettler Toledo TGA 2 Star System equipment (Mettler Toledo, Columbus, OH, USA), in the range 30 to 1000 °C, using approximately 5 mg of sample, with a heating rate of 10 °C/min, under a nitrogen atmosphere and gas flow of 50 mL/min, in an alumina crucible.

The gel content of SBR was determined in triplicate, following the ASTM D 3616 standard. The analysis was conducted using Soxhlet extraction to extract the soluble fraction, using toluene and 1 g of sample, for 24 h. Then, the residual material was dried in a vacuum oven at 60 °C for 24 h. The procedure to calculate the gel content of the SBR powder followed the method described in the literature [32], being determined by Equations (1) and (2):

(1) $$\%\mathrm{EPM}=\frac{\%M_{e}}{100-{\%M}_{r}}\times 100$$

Gel content = 100 − (%EPM)(2)

where %EPM = % extraction of polymeric material; %Me = soluble fraction determined in the Soxhlet extractor; %M_r_ = residual fraction obtained by TGA.

Scanning electron microscopy (SEM) analysis was performed on the surface of the SBR powder using a VEGAN 3 TESCAN microscope operating at 10 kV and under a high vacuum (TESCAN, Brun, Czech Republic). The sample surface was coated with gold.

### 2.4. Characterization of Compounds

The rheological curves were obtained using an internal mixer (Thermo Scientific, Waltham, MA, USA) equipped with roller-type rotors, operating at 190 °C and a rotation speed of 60 rpm, in an air atmosphere for 10 min.

The melt flow index (MFI) was measured using a plastometer (Hebert Lambert, São Paulo, Brazil), according to ASTM D1238 standards, with a 2.16 kg load at 230 °C. The results were obtained from an average of eight samples.

The electrical conductivity (σ) test was conducted on compression-molded films with a thickness of approximately 0.5 mm. The measurements were taken using a Keithley electrometer (model 8009) and the volumetric method (Keithley Instruments, Cleveland, OH, USA), with a current of 20 mA and a voltage of 1 volt.

The impact strength test was conducted on notched specimens following ASTM D256. The test was performed at room temperature using a Ceast Resil 5.5 J apparatus, equipped with a 2.75 J hammer (Instron, Norwood, MA, USA). Results were based on the average of eight specimens.

Tensile tests were conducted on injection-molded specimens following ASTM D638, using a universal testing machine from Oswaldo Filizola BME with a 20 kN load cell and a 50 mm/min loading rate (Oswaldo Filizola Ltda, São Paulo, Brazil). The tests were performed at room temperature, and the results were based on the average of eight specimens.

The Shore D hardness test was conducted in accordance with ASTM D2240, using a 50 N load controlled by calibrated springs (MetroTokyo, São Paulo, Brazil). The results were determined based on the average of eight measurements.

Differential scanning calorimetry (DSC) was performed using a Shimadzu DSC-60Plus instrument (Shimadzu Corporation, Kyoto, Japan) with nitrogen as the carrier gas at a flow rate of 50 mL/min. The temperature was scanned from 30 °C to 200 °C (heating–cooling–heating) at a rate of 10 °C/min, using 3 mg of sample. The degree of crystallinity (Xc) was determined according to Equation (3):

(3) $$X_{c}=\frac{{∆H}_{f}}{w*{∆H}_{f100\%}}*100\%$$

where ∆H_f_ = enthalpy of crystalline melting obtained by DSC; w = mass fraction of PP; ∆H_f100%_ = enthalpy of melting of PP with 100% crystallinity, 207 J/g [33].

The heat deflection temperature (HDT) was determined following ASTM D648, using a Ceast HDT 6 VICAT apparatus (Instron, Norwood, MA, USA). The test was conducted with a load of 1.82 MPa and a heating rate of 120 °C/h (method A). The temperature at which the sample exhibited a deflection of 0.25 mm was recorded. Results were based on the average of three specimens.

The morphology was examined on the fracture surfaces of impact-tested samples using a VEGAN MIRA LMU scanning electron microscope (SEM) (TESCAN, Brun, Czech Republic) with a voltage of 5 keV and in high vacuum. The sample surfaces were coated with gold.

## 3. Results and Discussion

### 3.1. Characterization of SBR

**Figure 1:**
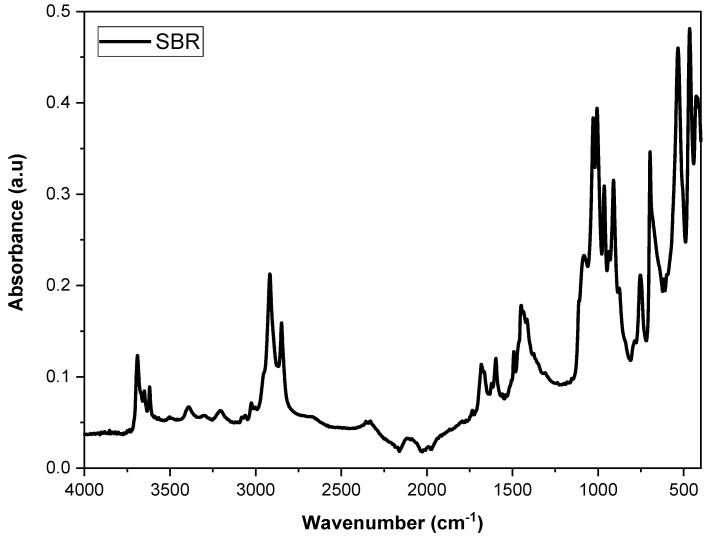
Spectrum of SBR powder from the shoe industry.

Figure 1 displays the FTIR spectrum of the SBR powder, highlighting the characteristic bands of styrene–butadiene rubber. The bands at 729 cm^−1^ and 965 cm^−1^ correspond to the cis-butadiene and trans-butadiene groups, respectively [34]. Additionally, the bands observed at 3023 cm^−1^, 2920 cm^−1^, and 2847 cm^−1^ are attributed to the stretching of CH groups in the aromatic rings of styrene, while the band at 697 cm^−1^ is associated with out-of-plane CH stretching [35,36]. The bands around 1029 cm^−1^ and 1006 cm^−1^ indicate the presence of C-S bonds, confirming that the SBR is vulcanized [37].

Particle size analysis was performed to determine the size distribution of the SBR particles after passing through a 60-mesh sieve (250 µm). The mechanical grinding process produced a powder with a predominance of larger particles: 69.4% in the range of 249–180 µm, 22.3% in the range of 179–150 µm, 6.7% in the range of 149–120 µm, and 1.6% smaller than 120 µm. Massarotto et al. [38] reported a similar particle size distribution.

**Figure 2:**
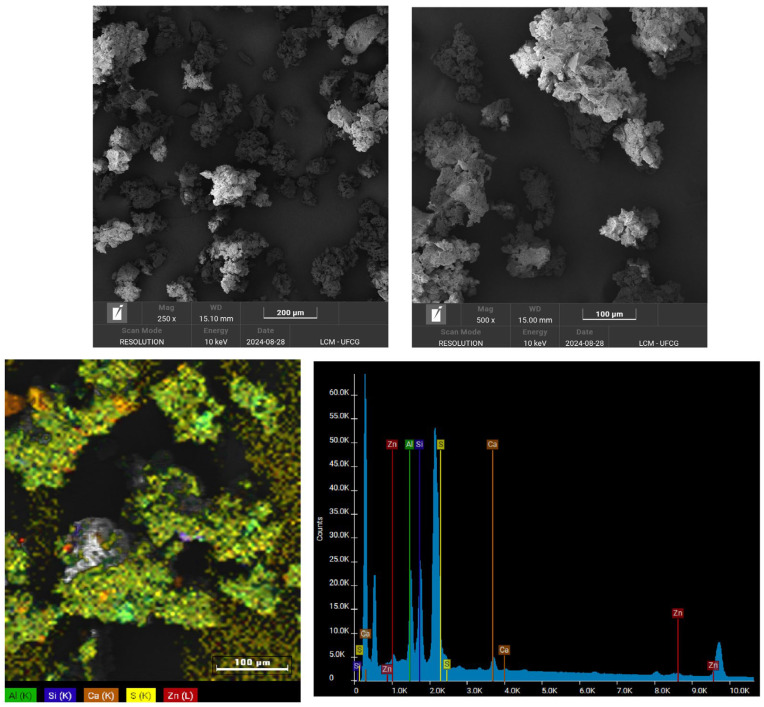
Morphology obtained by SEM of SBR powder, with magnifications of 250× and 500×.

Figure 2 shows the surface morphology of the SBR powder as observed by SEM, with magnifications of 250× and 500×. The SBR powder exhibits a rough and irregular surface, a characteristic resulting from the mechanical grinding process. Additionally, the SEM images reveal a heterogeneous particle size distribution and the presence of agglomerates, which aligns with the previously reported granulometric distribution. Similar morphological features in rubber powder have been reported in the literature [39]. Regarding the mapping and the EDS spectrum, the elements aluminum (Al), silicon (Si), calcium (Ca), sulfur (S), and zinc are observed, possibly associated with kaolin, calcium carbonate, and zinc oxide, as demonstrated below in the EDX.

The rubber used in the shoe industry has a complex composition due to incorporating numerous additives that enhance the product formulation [40]. Active and inactive mineral fillers are typically added to improve properties and reduce costs. The SBR powder was calcined at 600 °C for 5 h to identify and quantify these additives. Table 2 presents the EDX results of the SBR following the calcination process. The presence of Al_2_O_3_ and SiO_2_ is likely associated with kaolin (Al_2_O_3_·2SiO_2_·2H_2_O), a filler commonly used to lower costs. CaO is probably linked to calcium carbonate, another filler [41]. Sulfur trioxide (SO_3_) is likely an impurity within the kaolin [42]. Zinc oxide (ZnO) plays an important role in controlling vulcanization, acting as an activator [43]. Additionally, titanium dioxide (TiO_2_) is present in low concentrations to mitigate the effects of photo-oxidative aging [44].

**Figure 3:**
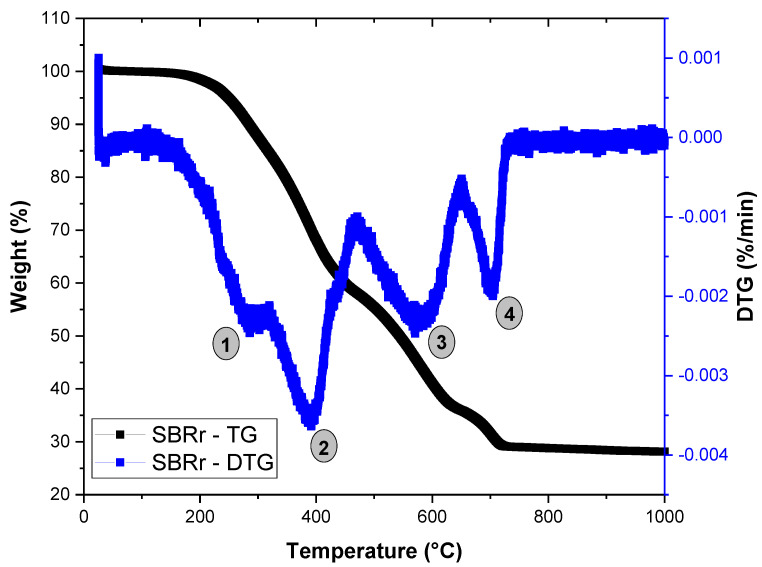
Thermogravimetric (TG) behavior of SBR powder.

Figure 3 presents the thermogravimetry (TG/DTG) curve of the SBR powder, used to assess the thermal stability of the rubber waste. The SBR exhibited a complex decomposition pattern with multiple mass loss events, consistent with the literature [45]. The initial decomposition occurred between 150 and 300 °C, corresponding to the evaporation of volatile organic additives, particularly the extender oil commonly used in rubber formulations to enhance processing [46]. A second degradation phase, occurring between 300 and 470 °C, is attributed to the scission of the styrene–butadiene (SBR) component in the elastomer. Between 470 and 650 °C, decomposition of calcium carbonate is likely, resulting in the release of CO_2_ and the formation of CaO [47], which aligns with the EDX chemical analysis results. The SBR residue used has a black hue due to the carbon black, which led to the mass loss event between 650 and 730 °C [48]. Additionally, a residue of 28.2% associated with mineral fillers was observed at 1000 °C.

The gel content of SBR was calculated using Equations (1) and (2), based on the 28.2% residue identified at 1000 °C in the TG analysis. This results in 89.1% crosslinked material and 10.9% soluble fraction. The gel content of 89.1% for SBR falls within the typical 85–93% range for vulcanized rubber, as reported in the literature [45,49,50].

### 3.2. Characterization of Compounds

**Figure 4:**
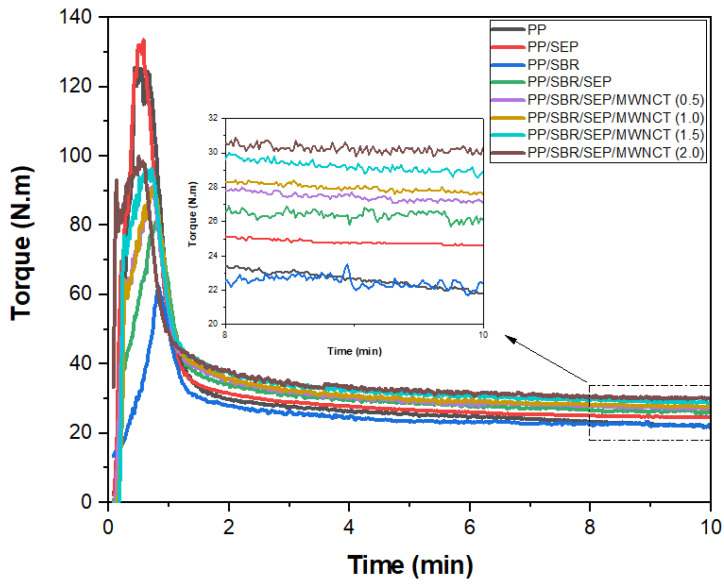
Torque rheometry curves for pure PP, PP/SEP, PP/SBR, and PP/SBR/SEP compounds, as a function of MWCNT content.

Figure 4 displays the torque rheometry curves for pure PP and polymeric compounds as a function of MWCNT content. During the 0–2 min processing interval, a maximum torque peak was observed, associated with the dissipation of mechanical energy. Subsequently, the torque decreased and stabilized due to the plasticization of the material, allowing the molten material to flow within the mixing chamber. This indicates that the torque is proportional to the viscosity. Pure PP and the PP/SEBS blend exhibited the highest maximum torque peak values, ranging from 120 to 140 N·m, reflecting a greater demand for mechanical energy to compact the solid material. In contrast, the PP/SBR, PP/SBR/SEP, and PP/SBR/SEP/MWCNT systems showed reduced maximum torque peaks, ranging from 60 to 100 N·m. This reduction suggests that SBR, being an elastomeric and flexible material, facilitated the compaction of PP during mechanical mixing.

The terminal torques at 10 min were 21.8 N·m for pure PP and 24.6 N·m for the PP/SEP blend. The increase in torque for the PP/SEP blend can be attributed to two factors: (1) the lower fluidity of SEP, which leads to higher torque, and (2) interactions between similar chemical groups, such as the propylene components of SEP and the PP matrix, resulting in increased molecular entanglement and, consequently, higher torque and viscosity. Despite SBR being a vulcanized residue, the incorporation of 30% by weight into PP did not significantly alter the terminal torque (22.4 N·m). Thus, the addition of 30% SBR did not adversely affect the processability of PP. When the PP/SBR compound was compatibilized with 10% SEP, the torque increased to 26.1 N·m, surpassing that of pure PP, the PP/SEP blend, and the PP/SBR system. This suggests that SEP acted as a compatibilizing agent for the PP/SBR compound, as part of its molecular structure interacts with both the PP and SBR components. The solubility parameter for PP is 16.6 (J/cm^3^)^1/2^, while SBR has a solubility parameter of 17.6 (J/cm^3^)^1/2^. The SEP copolymer exhibits solubility parameters of 17.6 (J/cm^3^)^1/2^ for styrene, 16.2 (J/cm^3^)^1/2^ for ethylene, and 16.6 (J/cm^3^)^1/2^ for propylene [51]. Consequently, in the PP/SBR/SEP compound, the interaction mechanism suggests that the propylene and styrene components of SEP interact with PP and SBR, respectively, leading to robust molecular entanglements. This increased interaction results in higher torque during processing, which enhances compatibility and improves impact strength and elongation at break, as will be discussed further. In the PP/SBR/SEP/MWCNT compounds, torque increased continuously with higher MWCNT content. The PP/SBR/SEP/MWCNT compound with 2 phr of MWCNT exhibited a terminal torque of approximately 30.3 N·m, reflecting a 16.1% increase compared to the PP/SBR/SEP compound. The MWCNTs, being nanofillers with a high surface area (>233 m^2^/g), enhance the viscosity of the PP/SBR/SEP system. Additionally, the presence of MWCNTs increases particle–particle friction within the molecular chain, leading to improved electrical conductivity.

**Figure 5:**
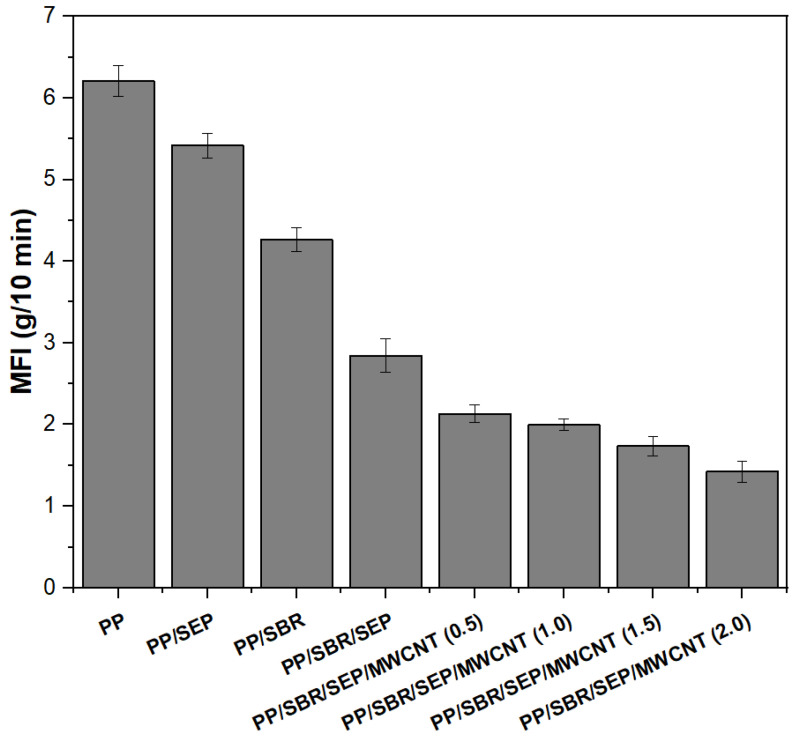
Melt flow index (MFI) for pure PP and polymeric compounds, with different MWCNT contents.

The melt flow index (MFI) is commonly used in the plastics industry to assess the flow characteristics of polymers. In Figure 5, the MFI was employed to measure the flow resistance of pure PP and polymer compounds, both with and without carbon nanotubes (MWCNT).

Pure PP exhibited the highest melt flow index (MFI) value of 6.2 g/10 min, indicating lower viscosity. Adding 10% SEP to the PP matrix reduced the MFI to 5.4 g/10 min, suggesting interactions between the propylene in SEP and the PP matrix that increase viscosity. The PP/SBR compound displayed an MFI of 4.2 g/10 min, reflecting greater flow resistance than pure PP. This increase in flow resistance is attributed to SBR, a vulcanized residue that does not flow or melt, thereby acting as a filler and restricting the flowability of PP. A different behavior was observed between the PP/SEP and PP/SBR systems. In the torque rheometry, the PP/SEP system exhibited higher viscosity, as evidenced by the higher torque. This suggests that the PP/SBR compound was more sensitive to severe shear conditions, such as those in the internal mixer, resulting in lower viscosity. The MFI decreased significantly for the PP/SBR/SEP compound, with a value of 2.8 g/10 min. This indicates more effective molecular entanglement and greater flow resistance. This result suggests that SEP enhanced the compatibility of the PP/SBR compound, leading to materials with improved flexibility. The addition of MWCNTs further increased the viscosity of the PP/SBR/SEP compounds, with MFI values ranging from 1.3 to 2.2 g/10 min. The MWCNT contents of 0.5 and 1 phr in the PP/SBR/SEP system produced MFI of 2.11 g/10 min and 1.98 g/10 min, which were close results considering the experimental error margin. Higher concentrations of 1.5 and 2 phr led to a continuous decrease in MFI compared to the PP/SBR/SEP system, which aligns with the torque rheometry results. As the MWCNT content increased, greater particle–particle and particle–polymer interactions likely occurred, resulting in increased friction in the polymer chain. This increases the material’s viscosity, implying a lower flow index.

Table 3 shows the electrical resistivity (ρ) and electrical conductivity (σ) results for pure PP and polymeric compounds with different MWCNT contents.

PP is an insulating material, but incorporating carbon-based nanofillers can enhance electrical conductivity, broadening the potential applications for electrically conductive products [52]. Materials are classified based on their electrical conductivity into categories such as insulators (10^−12^ to 10^−22^ S/cm), semiconductors (10^−02^ to 10^−09^ S/cm), conductors (10^2^ S/cm), or superconductors (10^20^ S/cm) [53,54]. Table 3 shows that PP, PP/SEP, PP/SBR, and PP/SBR/SEP exhibited high resistivity, indicating low electrical conductivity and insulating behavior. Adding 0.5 phr and 1 phr of MWCNTs did not enhance electrical conductivity in the PP/SBR/SEP system, considering that the order of magnitude was maintained at 10^−11^ S/cm. With the increase in MWCNT content to 1.5 phr and 2 phr, a significant reduction in electrical resistivity was observed, indicating the formation of an electrical percolation network. Specifically, the PP/SBR/SEP/MWCNT (1.5) and PP/SBR/SEP/MWCNT (2.0) compounds transitioned into the semiconductor material category, with conductivities in the range of 10^−02^ to 10^−09^ S/cm. For antistatic applications, flexible materials are required to have electrical conductivity greater than 10^−08^ S/cm [55]. According to Pan et al. [56] and Silva et al. [57], materials with volumetric electrical resistivity in the order of 10^06^ exhibit electrostatic dissipative behavior, with potential for antistatic applications. Therefore, the PP/SBR/SEP/MNWCNT (2.0) compound with ρ = 2.21 × 10^06^ Ω.cm and σ = 4.52 × 10^−07^ S/cm has the potential to dissipate static charges. Antistatic materials are widely utilized in industries that handle electronic components and packaging for sensitive products. Additionally, they have potential applications in coating materials to prevent the accumulation of static electricity [58].

**Figure 6:**
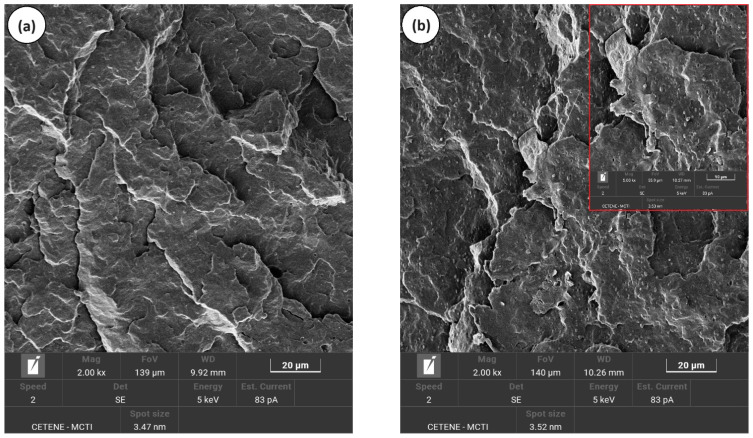

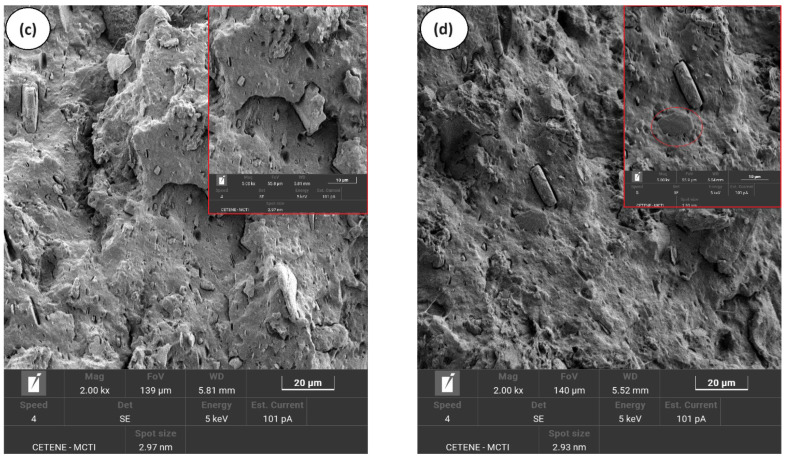
Morphology obtained by SEM, for (**a**) pure PP; (**b**) PP/SEP; (**c**) PP/SBR; (**d**) PP/SBR/SEP.

Figure 6a–d shows the SEM morphology for pure PP, PP/SEP, PP/SBR, and PP/SBR/SEP, with magnifications of 2000× and 5000×. In Figure 6a, PP presented an irregular fracture with plastic deformation, suggesting ductile material behavior. Incorporating 10% SEP into the PP matrix resulted in a biphasic morphology characteristic of an immiscible blend. SEP was well-dispersed in the PP, making it effective as an impact modifier. Additionally, the size of the SEP droplets was refined during mixing with PP due to the favorable interaction between the phases, as discussed in the torque rheometry analysis. During the impact test, SEP retained most droplets that adhered to the PP, as shown in Figure 6b. The SEP droplets were not pulled out, indicating good interfacial adhesion between the PP and SEP. It appears that the propylene fraction in SEP effectively stabilizes and enhances the interaction with the PP, resulting in reduced interfacial tension between the components. This improved the interface resistance and contributed to a more effective deformation mechanism, as demonstrated subsequently. Similar behavior has been reported in the literature by Abreu et al. [59], who observed analogous effects in the development of PP/SEBS and PP/SBS blends. Figure 6c shows that the PP/SBR compound exhibited a coarse morphology, characterized by both large and small particles, poor interfacial adhesion, and many voids. The differing molecular structures of PP and SBR do not promote specific interactions, resulting in the observed coarse morphology. The PP/SBR/SEP system did not significantly modify morphology in relation to PP/SBR. However, it is possible to notice a smaller amount of voids and, at the same time, large particles with interface connections (Figure 6b—5000× magnification). This suggests that SEP promoted an interfacial interaction with PP/SBR, contributing to the adhesion of large particles in the PP matrix. Some SBR phases still have low adhesion to PP, suggesting that not all SEP migrated to the interface. Small voids are visualized in PP (see Figure 6d), possibly due to the SEP that was pulled out during the impact test. The refinement of the dispersed phase in the PP/SBR/SEP system may have resulted from two conditions: either the breakage of SBR particles during processing or a fraction of the SEP remained dispersed in the PP matrix, forming a third phase, as also reported in the literature [60]. Consequently, the SEP also contributed to the toughening of the PP, generating the good impact strength results presented later.

**Figure 7:**
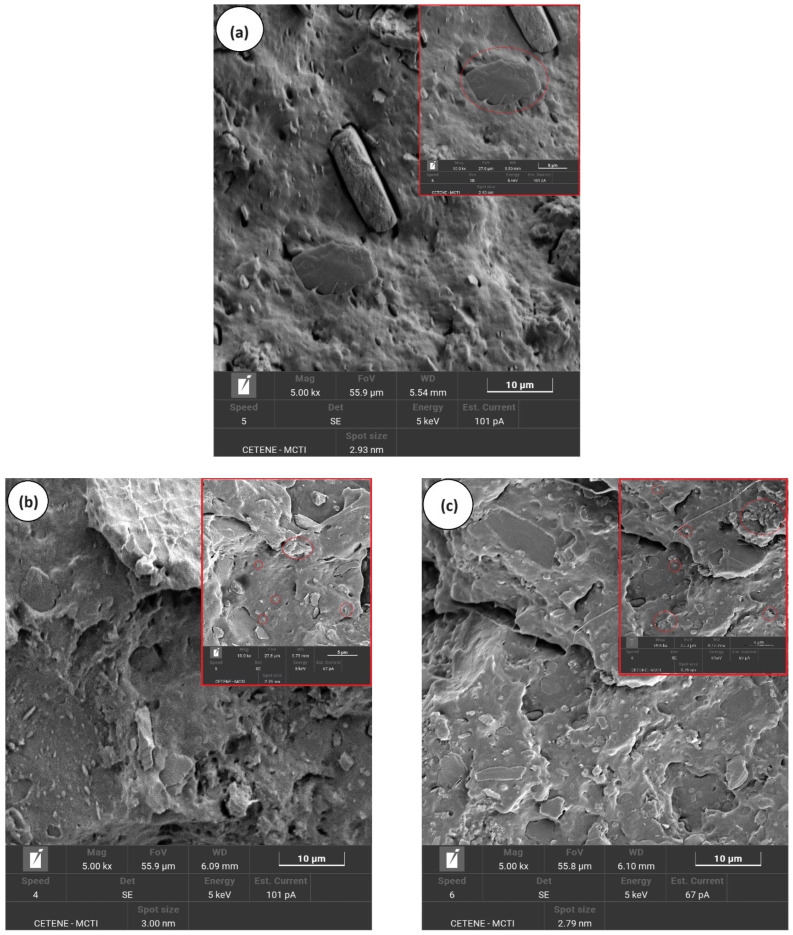

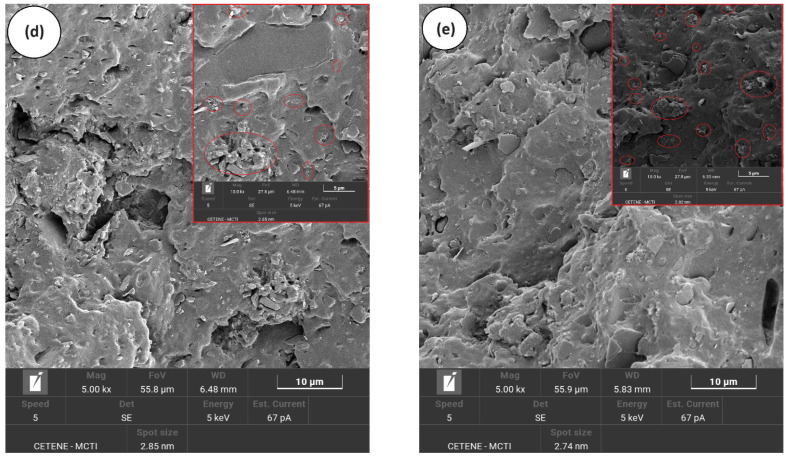
Evolution of the morphology by SEM, for (**a**) PP/SBR/SEP; (**b**) PP/SBR/SEP/MWCNT (0.5 phr); (**c**) PP/SBR/SEP/MWCNT (1.0 phr); (**d**) PP/SBR/SEP/MWCNT (1.5 phr); (**e**) PP/SBR/SEP/MWCNT (2.0 phr).

Figure 7a–e illustrates the evolution of the morphology in the PP/SBR/SEP system with varying MWCNT contents, shown at magnifications of 5000× and 10,000×. The PP/SBR/SEP and PP/SBR/SEP/MWCNT samples display a hybrid distribution of the dispersed phase, characterized by small and large particles. In Figure 7a, the PP/SBR/SEP compound exhibits large particles with connections in the interfacial region, indicating that SEP migrated to the interface between PP and SBR. This is significant because it enhances stress transfer between the phases and promotes more efficient energy dissipation in the matrix during the impact test, as demonstrated subsequently. At the same time, dispersed particles with poor interfacial adhesion and small cavities were present, likely due to a fraction of the SEP remaining dispersed in the PP as a third phase, contributing to the toughening mechanism. Apparently, the incorporation of MWCNT into the PP/SBR/SEP system improved the distribution process of the SBR in the PP matrix, as shown in Figure 7b–e. Carbon nanotubes can act as compatibilizers, enhancing the distribution of the phase dispersed, as demonstrated in the study by Wu et al. [61]. As the MWCNT content in PP/SBR/SEP increased, there was a tendency to stabilize the morphology, with particles distributed and adhered to the PP matrix. The behavior was more prominent in the morphology for the PP/SBR/SEP/MWCNT (1 phr) compound, given the adhered and distributed particles, which reflected in the better mechanical behavior under impact. The literature [62,63] reported that carbon nanotubes reduce interfacial tension in polymer blends by stabilizing the dispersed phase through steric hindrance. This minimizes collision and agglomeration of the dispersed phase during processing, generating a more compatible morphology. However, from the point of view of carbon nanotubes in the PP/SBR/SEP system, there was a tendency for greater distribution in the PP matrix for higher MWCNT content. For MWCNT contents of 0.5 phr and 1 phr, the distribution of nanotubes was uneven, with concentrations in some areas and none in others (see Supplementary Material S1). However, with increased MWCNT contents of 1.5 phr and 2 phr, there was a greater amount of MWCNT in the PP matrix. This was particularly evident in the PP/SBR/SEP/MWCNT (2 phr) compound, which favored higher electrical conductivity. In Supplementary Material S1 (SEM with 20,000× magnification), the formation of carbon nanotube agglomeration in the PP matrix was observed, particularly for 2 phr. This finding promoted a more severe deleterious effect on the mechanical properties, resulting in reduced ductility, as discussed further below.

**Figure 8:**
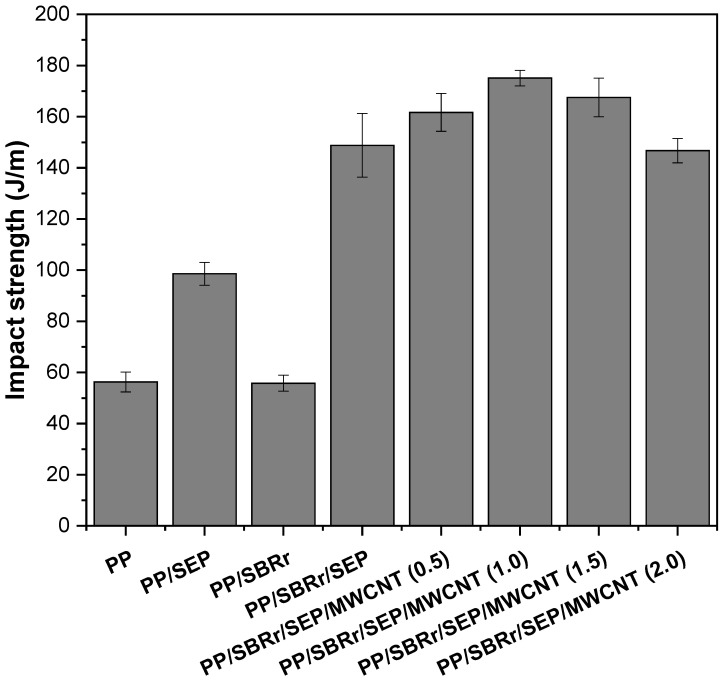
Impact strength for pure PP and polymeric compounds, with and without carbon nanotubes.

Figure 8 shows the impact strength results for pure PP and polymeric compounds as a function of MWCNT concentration. Pure PP had an impact strength of approximately 57 J/m, while the PP/SEP blend increased this value to 98.5 J/m. The addition of 10% SEP acted as an impact modifier for PP, enhancing energy dissipation during the impact test. SEM analysis revealed that the PP/SEP blend exhibited good dispersion and small droplet size, contributing to improved toughness. In contrast, the PP/SBR compound did not improve impact strength compared to pure PP. Despite the addition of 30% SBR to the PP matrix, there was no significant toughening effect due to the differences in molecular structure between PP and SBR. Consequently, stress transfer was inefficient in the interfacial region between PP and SBR, resulting in a slight decrease in impact strength to 54.3 J/m.

The PP/SBR/SEP compound significantly improved the impact strength to 148.9 J/m, surpassing the impact strength of high-impact polystyrene (100–120 J/m), which is commonly used in applications such as toys, housewares, and furniture accessories [64]. In percentage terms, the PP/SBR/SEP compound exhibited a 161.2% increase compared to pure PP and a 174.2% increase compared to PP/SBR. The SEP copolymer enhanced the compatibility of PP and SBR, producing a synergistic effect on impact properties, as supported by the torque rheometry and MFI results. This suggests that SEP facilitated interactions between PP and SBR, optimizing stress transfer and improving energy dissipation in the PP matrix. Furthermore, as previously discussed, a fraction of the SEP possibly remained dispersed in the PP matrix, contributing to the improved toughness. Interestingly, the impact strength increased by adding a small amount of MWNCT to the PP/SBR/SEP compound. For example, the PP/SBR/SEP/MWCNT (0.5 phr) compound improved the impact strength to 161.7 J/m. Further increasing the MWCNT content to 1 phr resulted in an impact strength of 175.1 J/m, corresponding to a 17.6% improvement over the PP/SBR/SEP system. However, at MWCNT concentrations of 1.5 phr and 2 phr, the impact strength began to decrease. The PP/SBR/SEP/MWCNT (1.5 phr) and PP/SBR/SEP/MWCNT (2 phr) compounds remain tough at room temperature, exceeding the impact strength of pure PP. The concentration of 1 phr MWCNT is critical for enhancing the impact strength of the PP/SBR/SEP compound; higher concentrations resulted in a detrimental effect on this property. This behavior can be attributed to the increase in MWCNT agglomerates at the 1.5 phr and 2 phr contents in the PP matrix, as demonstrated in the morphology obtained by SEM in Supplementary Material S1. Consequently, there was a higher stress concentration, which led to a decline in the impact strength for the PP/SBR/SEP/MWCNT (1.5 phr) and PP/SBR/SEP/MWCNT (2 phr) compounds, compared to the compositions containing 0.5 phr and 1 phr. In contrast, MWCNT contents of 0.5 phr and 1 phr enhanced the toughening mechanism of the PP/SBR/SEP compound, possibly by improving morphological stability or acting as a barrier to crack propagation during impact testing [62].

**Figure 9:**
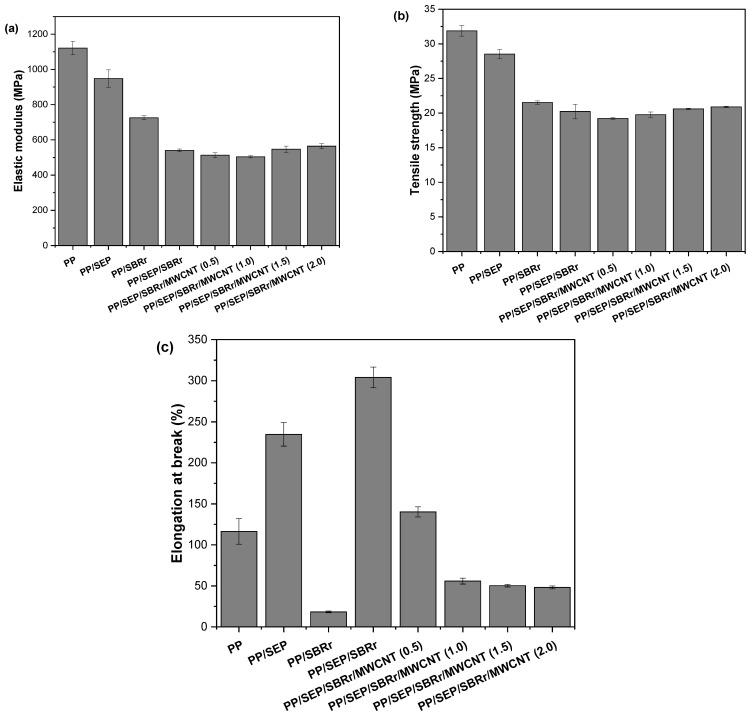
Mechanical properties under tensile for (**a**) elastic modulus; (**b**) tensile strength; (**c**) elongation at break.

Figure 9a–c presents the results of the tensile mechanical tests for pure PP, PP/SEP, PP/SBRr, PP/SBRr/SEP, and PP/SBRr/SEP/MWCNT compounds. Figure 9a shows that pure PP exhibits the highest elastic modulus, approximately 1121 MPa. The addition of SEP and SBRr leads to a reduction in material stiffness. This decrease is attributed to the increased flexibility imparted by the elastomeric properties of SEP and SBR, which results in a lower elastic modulus. The PP/SBR/SEP system exhibited a more pronounced decrease in stiffness due to the higher proportion of elastomeric components (30% SBR and 10% SEP). Incorporating carbon nanotubes (1.5 phr and 2 phr) into the PP/SBR/SEP system led to a slight increase in the elastic modulus, though the gain was not substantial. A similar trend was observed for the maximum stress. The addition of SEP and SBRr reduced the maximum stress of the materials compared to pure PP. The polymeric compounds deform under lower stresses due to the elastic behavior of SEP and SBR. The PP/SBR/SEP system exhibited a more significant reduction in tensile strength, attributed to its increased flexibility, as confirmed by impact resistance tests. For the PP/SBR/SEP/MWCNT compounds, a slight improvement in tensile strength was observed with higher MWCNT content compared to PP/SBR/SEP. However, considering the experimental error margin, the results are close.

Elongation at break is a terminal mechanical property that depends on good interaction between the components to maximize this property. Figure 9c shows a significant increase in elongation at break for the PP/SEP and PP/SBRr/SEP systems compared to pure PP. The deformation mechanism was notably enhanced in the PP/SBRr/SEP compound, with elongation approaching 300%. This supports the hypothesis that SEP acts as a compatibilizing agent for PP/SBR, thereby improving ductility. Additionally, in the PP/SEP blend, the SEP propylene group facilitates interactions with the polymer matrix, improving elongation at break. The PP/SBRr/SEP compound demonstrated an improved deformation mechanism because SEP contains chemical groups that interact with both PP and SBR, leading to better compatibility and an increase in elongation at break. However, for the PP/SBRr/SEP/MWCNT compounds, elongation at break decreased gradually with higher MWCNT concentrations. This behavior is attributed to MWCNTs being nanofillers that restrict mobility and adversely affect the deformation mechanism. Furthermore, as shown in the Supplementary Material S1, carbon nanotube agglomerates formed in the PP matrix, especially for 1 phr, 1.5 phr, and 2 phr. This results in stress concentration during mechanical testing under tensile, which promotes crack nucleation and hinders the ductile deformation mechanism. Consequently, this leads to premature failure during elongation at break.

**Figure 10:**
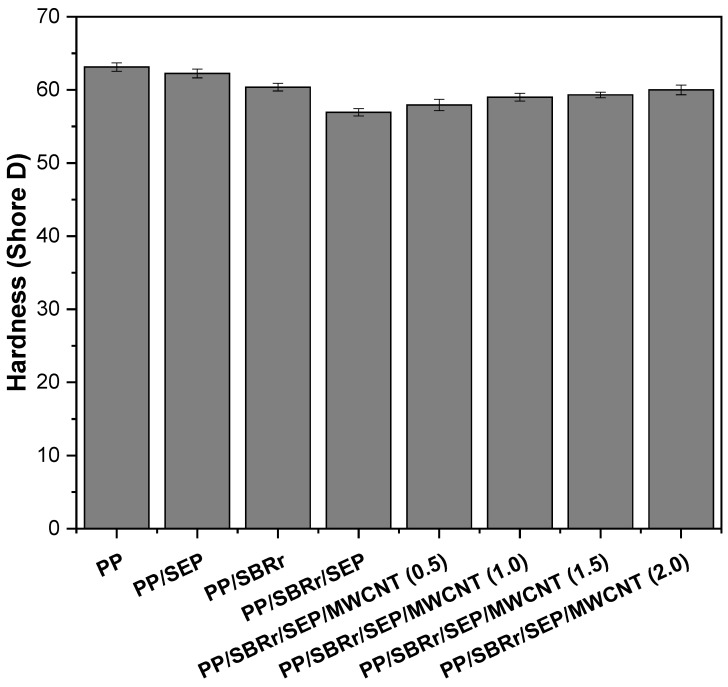
Shore D hardness for pure PP and polymeric compounds, as a function of MWCNT content.

Figure 10 presents the Shore D hardness results for pure PP and polymeric compounds with varying MWCNT contents. Pure PP exhibits a hardness of approximately 63.1 Shore D, which aligns with values reported in the literature [65]. The blends containing 10% SEP and 30% SBRr show a slight decrease in Shore D hardness, with values of 62.2 and 60.3, respectively. This reduction is due to the addition of elastomeric materials to the polypropylene matrix, which lowers the surface hardness compared to pure PP. The PP/SBRr/SEP compound exhibited a hardness of 56.9 Shore D, which is lower than that of the PP/SBR system. This decrease is likely due to the higher proportion of elastomeric materials (30% SBRr and 10% SEP), which enhances flexibility. The incorporation of MWCNTs into the PP/SBRr/SEP system resulted in a slight increase in surface hardness, reaching approximately 60 Shore D with 2.0 phr MWCNT. This suggests that the carbon nanotubes, distributed in the PP/SBRr/SEP compound, contributed to a moderate increase in surface stiffness. An increase in surface hardness with the addition of carbon nanotubes has also been reported in the literature [66,67].

**Figure 11:**
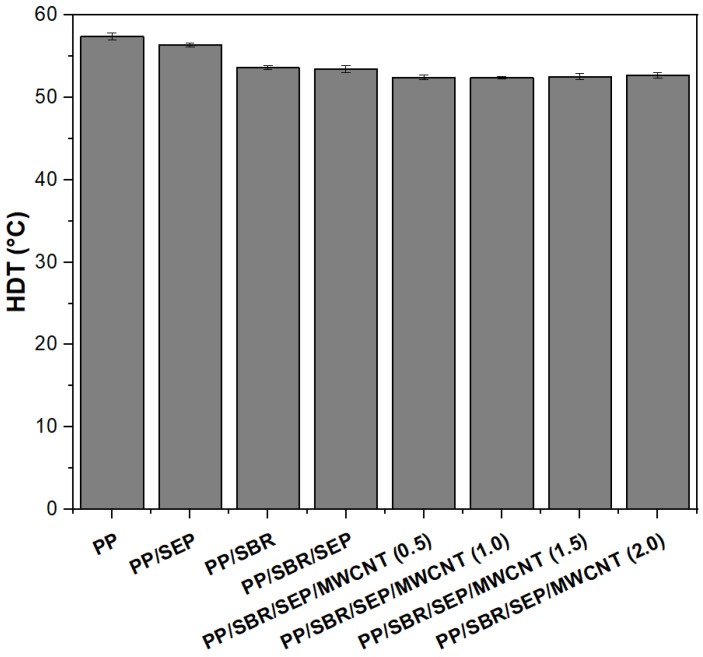
HDT results for pure PP and polymer compounds with different MWCNT contents.

Heat deflection temperature (HDT) is an important property for selecting materials in the polymer industry, as it indicates a material’s ability to maintain structural stability under load and temperature [68]. Figure 11 shows the HDT results for pure PP and various polymeric compounds. Pure PP exhibits the highest HDT, around 57.4 °C. The addition of 10% SEP to the PP matrix results in a slight decrease in HDT to 56.4 °C, due to the elastomeric nature of SEP. The introduction of SBRr further reduces the HDT to 53.6 °C, owing to the higher elastomeric content (30% by weight). The HDT of the PP/SBR/SEP system remains at 53.4 °C, similar to that of PP/SBR. Incorporating MWCNTs into the PP/SBRr/SEP system does not lead to significant changes in HDT with increased MWCNT content, suggesting that the low concentration of carbon nanotubes does not substantially improve HDT. As reported in the literature [69], the primary factors that significantly enhance HDT are high stiffness and a high degree of crystallinity. Although incorporating carbon nanotubes did increase the elastic modulus slightly, this improvement was not substantial enough to significantly boost the HDT. It appears that the high content of elastomeric materials (SBR and SEP) inhibited improvements in HDT due to the flexibility of these components.

**Figure 12:**
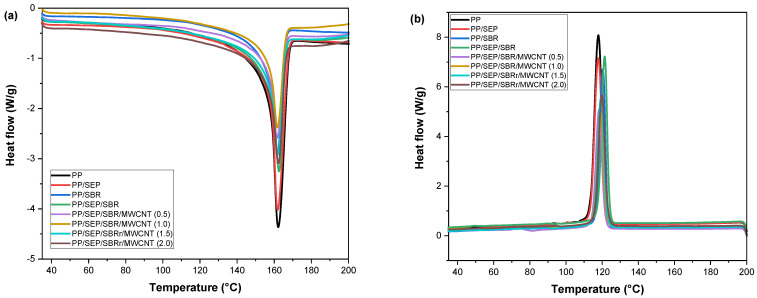
Curves obtained by DSC for pure PP, PP/SEP, PP/SBR, and composites compatibilized with and without carbon nanotubes, for (**a**) crystalline melting temperature; (**b**) crystallization temperature.

Figure 12a,b shows the DSC curves for the crystalline melting temperature (T_m_) and crystallization temperature (T_c_) obtained during the second heating cycle. The thermal properties as determined by DSC are summarized in Table 4.

The crystalline melting temperature (T_m_) of pure PP was 162.3 °C, consistent with values reported in the literature [70]. The PP/SEP and PP/SBR systems exhibited similar Tm values, comparable to that of pure PP. Adding SEP to the PP/SBR system caused a slight increase in T_m_ to 162.4 °C, in relation to PP/SEP and PP/SBR. The T_m_ for the PP/SBR/SEP/MWCNT compounds ranged from 161 to 162.5 °C, maintaining values similar to those of the PP matrix. Overall, the crystalline melting temperature behavior was maintained for the polymeric compounds. The peak crystallization temperature (Tc) for pure PP was 117.9 °C, while the PP/SEP system maintained a similar value of around 117.8 °C. Incorporating 30% SBR into PP increased T_c_ to 120 °C, indicating an accelerated crystallization process. This enhancement is likely due to the mineral fillers present in the SBR composition, as confirmed by EDX analysis. The crystallization process was further accelerated in the PP/SBR/SEP compound, with T_c_ shifting to 121.3 °C. This improvement is attributed to the better distribution of the dispersed phase with SEP, which facilitated the crystallization process. Adding MWCNTs to PP/SBR/SEP resulted in a slight reduction in T_c_, with values ranging between 118 and 120 °C. Nevertheless, the PP/SBR/SEP/MWCNT compounds exhibited a higher Tc than pure PP, indicating that crystallization begins at a higher temperature. The degree of crystallinity for pure PP was approximately 43.2%, which is typical for a semicrystalline material. The incorporation of SEP and SBR into the PP matrix reduced the degree of crystallinity, likely because these elastomers are predominantly amorphous, hindering crystallization. However, the PP/SBR/SEP compound exhibited an increased degree of crystallinity, reaching 44.1%, surpassing that of pure PP. This suggests that the SBR/SEP combination acted as a nucleating agent in the PP matrix, resulting in higher T_c_ and X_c_. Conversely, the PP/SBR/SEP/MWCNT compounds showed a more pronounced reduction in crystallinity compared to PP/SBR/SEP. Apparently, the carbon nanotubes inhibited the crystallization process and formation of stable nuclei of the PP/SBR/SEP system, generating a reduction in T_c_ and X_c_ values.

## 4. Conclusions

Vulcanized SBR residue is a raw material rich in additives, which generates the potential for reintroduction into the plastics transformation chain. One alternative is to use it as an impact modifier for polypropylene, provided SEP is a compatibilizing agent. The SEP copolymer interacted with PP and SBR, improving the degree of molecular entanglement, which increased viscosity. The PP/SBR/SEP compound showed ductile behavior, with high impact strength and elongation at break of around 300%. In addition, there were no severe losses in Shore D hardness and HDT. To expand the range of applications, incorporating carbon nanotubes in PP/SBR/SEP is attractive for producing flexible packaging with antistatic potential. The 2 phr concentration of MWCNT in the PP/SBR/SEP system maintained high impact strength at room temperature, with a conductive behavior of 4.52 × 10^−07^ S.cm and antistatic potential. The results presented are essential for plastics recycling, considering the possibility of developing antistatic polymeric compounds for electronic products.

## Figures and Tables

**Table 1:** Processed compositions of the compounds.

Samples	PP (wt%)	SBRr (wt%) *	SEP (wt%)	MWCNT (phr)
PP	100	-	-	-
PP/SEP	90	-	10	-
PP/SBR	70	30	-	-
PP/SBR/SEP	60	30	10	-
PP/SBR/SEP/MWCNT	60	30	10	0.5
PP/SBR/SEP/MWCNT	60	30	10	1.0
PP/SBR/SEP/MWCNT	60	30	10	1.5
PP/SBR/SEP/MWCNT	60	30	10	2.0

* The 30% SBR content was selected because it provided good injection molding.

**Table 2:** Chemical composition of SBR after calcination process at 600 °C.

Elements	Al_2_O_3_	SiO_2_	CaO	SO_3_	ZnO	TiO_2_	Others
Quantity (%)	45.8	38.6	4.4	4.2	3.1	1.7	2.2

**Table 3:** Resistivity and electrical conductivity of PP and polymeric compounds, as a function of MWCNT content.

Compounds	ρ (Ω.cm)	σ (S/cm)
PP	1.61 × 10^10^	6.21 × 10^−11^
PP/SEP	1.51 × 10^10^	6.62 × 10^−11^
PP/SBR	1.55 × 10^10^	6.45 × 10^−11^
PP/SBR/SEP	1.56 × 10^10^	6.41 × 10^−11^
PP/SBR/SEP/MNWCNT (0.5)	1.53 × 10^10^	6.53 × 10^−11^
PP/SBR/SEP/MNWCNT (1.0)	1.57 × 10^10^	6.37 × 10^−11^
PP/SBR/SEP/MNWCNT (1.5)	1.69 × 10^07^	5.92 × 10^−08^
PP/SBR/SEP/MNWCNT (2.0)	2.21 × 10^06^	4.52 × 10^−07^

**Table 4:** Thermal parameters obtained by DSC for the crystalline melting temperature (T_m_), crystallization temperature (T_c_) and the degree of crystallinity (X_c_).

Samples	T_m_ (°C)	T_c_ (°C)	X_c_ (%)
PP	162.3	117.9	43.2
PP/SEP	161.8	117.8	41.7
PP/SBR	161.4	120.4	40.9
PP/SBR/SEP	162.5	121.3	44.1
PP/SBR/SEP/MNWCNT (0.5)	161.5	118.2	35.2
PP/SBR/SEP/MNWCNT (1.0)	161.8	118.9	37.2
PP/SBR/SEP/MNWCNT (1.5)	161.9	119.2	41.6
PP/SBR/SEP/MNWCNT (2.0)	162.2	119.7	37.0

## Data Availability

Data are contained within the article.

## Supplementary Materials

The following supporting information can be downloaded at: https://www.mdpi.com/article/10.3390/polym16172542/s1. Figure S1. Evolution of the morphology by SEM, for: (a) PP/SBR/SEP/MWCNT (0.5 phr); (b) PP/SBR/SEP/MWCNT (1.0 phr); (c) PP/SBR/SEP/MWCNT (1.5 phr); (d) PP/SBR/SEP/MWCNT (2.0 phr).
