# Supplementary material for: From Waste to Styrene–Butadiene (SBR) Reuse: Developing PP/SBR/SEP Mixtures with Carbon Nanotubes for Antistatic Application
Source: Polymers (Basel). 2024 Sep 8;16(17):2542. doi: 10.3390/polym16172542 (PMC11398230; doi:10.3390/polym16172542)
Supplement: Supplementary file 1 [file polymers-16-02542-s001.zip › polymers-3192731-supplementary.pdf]

From Waste to Styrene–Butadiene (SBR) Reuse: Developing PP/SBR/SEP Mixtures with Carbon Nanotubes for Antistatic Application

Edson Duarte de Melo Sobrinho [1], Eduardo da Silva Barbosa Ferreira [2], Flávio Urbano da Silva [2,3], Elieber Barros Bezerra [4], Renate Maria Ramos Wellen [4], Edcleide Maria Araújo [2] and Carlos Bruno Barreto Luna [2,*]

1 Academic Unit of Mechanical Engineering, Federal University of Campina Grande, Av. Aprígio Veloso, 882–Bodocongó, Campina Grande 58429-900, Paraíba, Brazil
2 Academic Unit of Materials Engineering, Federal University of Campina Grande, Av. Aprígio Veloso, 882–Bodocongó, Campina Grande 58429-900, Paraíba, Brazil; flavio.urbano@ifrn.edu.br (F.U.d.S.)
3 Federal Institute of Education, Ciência e Tecnologia do Rio Grande do Norte, Natal 59015-000, Rio Grande do Norte, Brazil
4 Department of Materials Engineering, Federal University of Paraíba, Cidade Universitária, João Pessoa 58051-900, Paraíba, Brazil
* Correspondence: brunobarretodemaufcg@hotmail.com

Supplementary material 1 (S1)

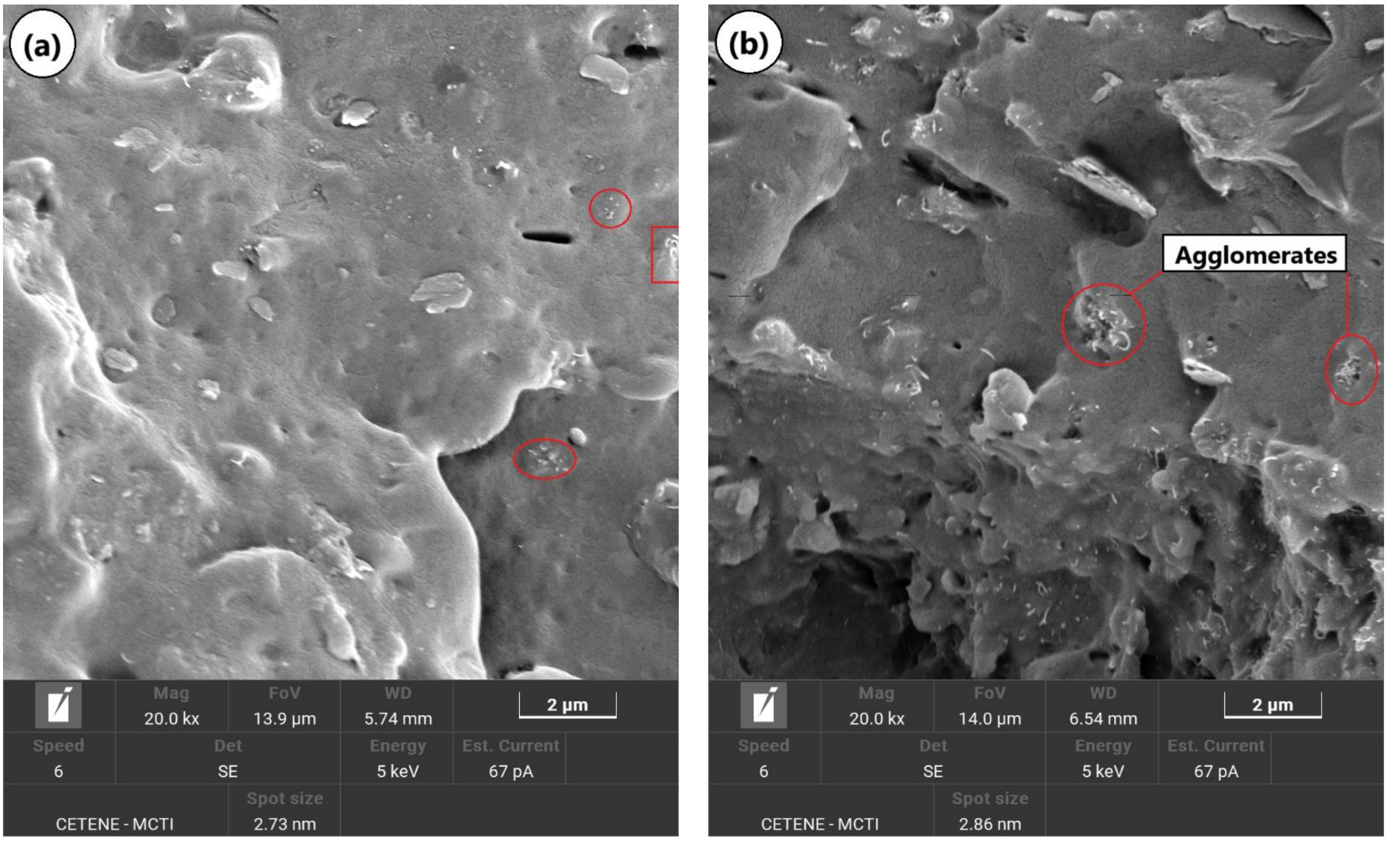

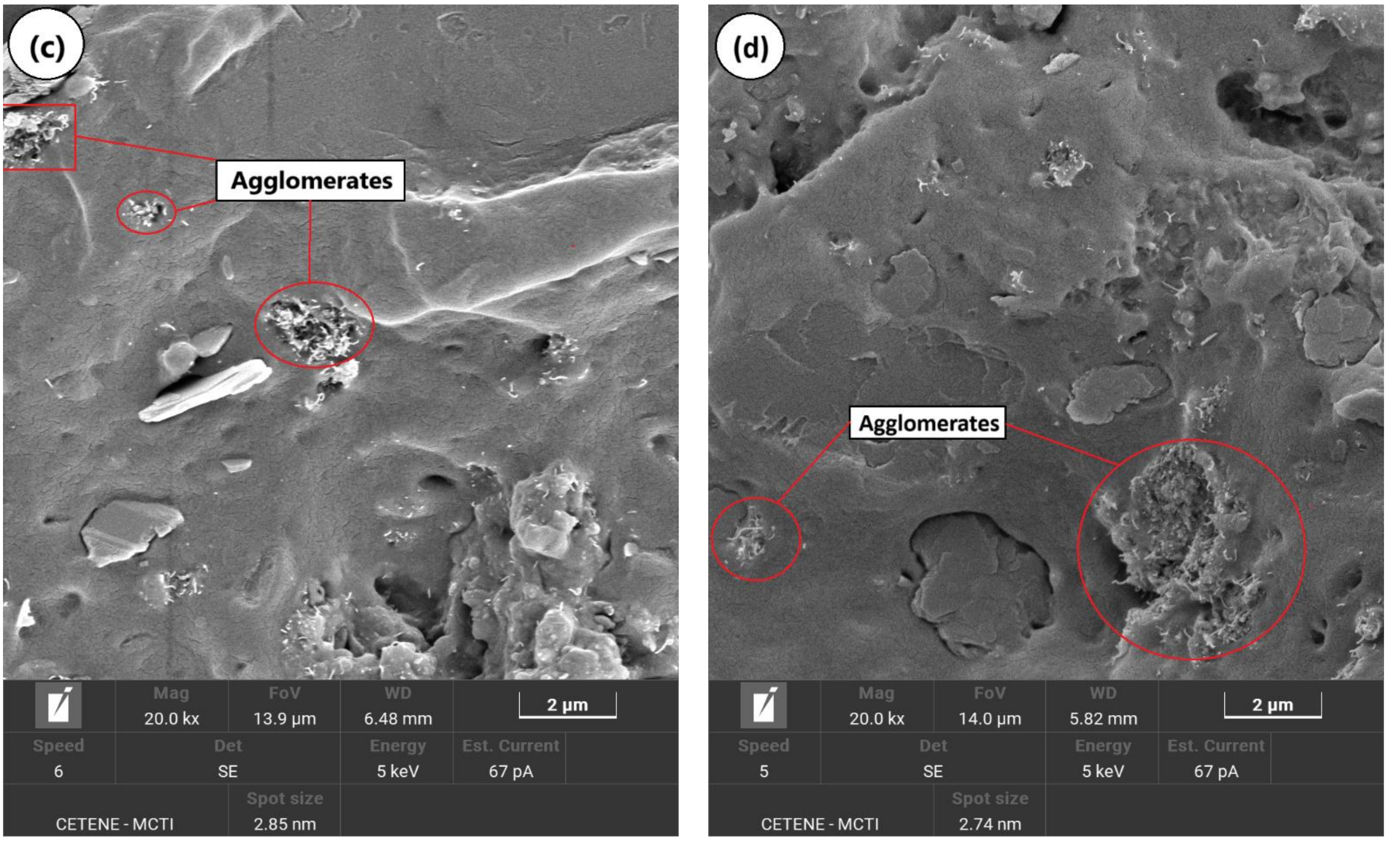

Figure S1. Evolution of the morphology by SEM, for: (a) PP/SBR/SEP/MWCNT (0.5 phr); (b) PP/SBR/SEP/MWCNT (1.0 phr); (c) PP/SBR/SEP/MWCNT (1.5 phr); (d) PP/SBR/SEP/MWCNT (2.0 phr).
